# A conversation with Peter Crompton, MD, MPH, Chief, Malaria Infection Biology and Immunity Section, National Institute of Allergy and Infectious Diseases

**DOI:** 10.1017/cts.2023.714

**Published:** 2023-01-17

**Authors:** Clinical Research Forum

**Affiliations:** Clinical Research Forum, Washington, DC, USA

## Top 10 Clinical Research Achievement Awards Q & A

This article is part of a series of interviews with recipients of Clinical Research Forum’s Top 10 Clinical Research Achievement Awards. This article is with Dr Peter Crompton, MD, MPH, Chief, Malaria Infection Biology and Immunity Section, National Institute of Allergy and Infectious Diseases. Dr Crompton studies malaria in Africa and he received a 2023 Top 10 Clinical Research Achievement Award for *Safety and Efficacy of a Monoclonal Antibody against Malaria in Mali. The interview has been edited for length and clarity.*

## How did you first become interested in medicine and infectious diseases?

Growing up, I learned about medicine from my grandfather, who was a small-town physician in northern Minnesota. After graduating from high school, I started traveling internationally and during college and medical school, I began doing public health-related projects on various infectious diseases in Africa and South America. I found infectious diseases to be fascinating scientifically but when I traveled, I saw firsthand their devastating effects on people and communities. I started to realize that maybe I could make a difference by focusing my medical career on the study of infectious diseases like malaria that disproportionally affect populations in low- and middle-income countries.

## When did you begin conducting research in Mali?

The observational studies and clinical trials we conduct on malaria in Mali are made possible through a close collaboration with an exceptional team of clinicians and scientists at the Malaria Research and Training Center at the University of Sciences, Techniques, and Technologies of Bamako (USTTB). I started this collaboration over 15 years ago when I was an infectious disease fellow at NIAID just getting started in research, and since then we have developed several field sites in Mali and very strong collaborative relationships with our colleagues at USTTB.

## How do you build strong collaborations like these?

There are two critical elements. The first involves the personal relationships and trust that I have developed with my colleagues at USTTB. They are world-class researchers who have always been welcoming to me and patient in teaching me about malaria--a disease that they have lived with their entire lives. From the start, it’s been a two-way street in terms of what I can bring to the table with research questions and biomedical technology and what they can teach me about doing field research on malaria and other infectious diseases in Africa. It’s this exchange of ideas that leads to the sense that weʼre trying to help each other as we work toward the common goal of defeating malaria. Iʼve worked with the same team in Mali for many years and we are now very close friends as well as colleagues. We’ve built a lot of trust in these relationships which I think is why when I approached them about conducting this phase 2 trial – the first study ever of a monoclonal antibody in Africa – they said, “Yes, let’s work together on this!”

The second element is the relationship between our research team and the study participants in the communities where we do our work. My colleagues at USTTB have a long-standing tradition of engaging the communities where we do research. For the most part, these are rural communities where there’s no modern electricity or plumbing, and where the residents grow their own food as subsistence farmers. Before we begin a research project, we meet with local leaders and representatives of these communities and spend a day or more answering their questions and addressing their concerns. From the very beginning of a new research study, the community is invited to participate and understand what’s going on. Then, we establish a research clinic and staff it 24/7 with young Malian doctors and nurses who are there to not only conduct the study but also to provide medical care to anyone in the village who needs it. Since 2011, we have been doing malaria studies in the community of Kalifabougou, Mali where we did the award-winning trial and so our research team has a strong relationship with the community. The residents of these communities are interested in the study results and there’s really a sense of pride that they’re contributing to the development of potential new tools that could be used to fight malaria across Africa. They are very much our partners in this work.

## What did the award-winning research show?

Our trial demonstrated that a single intravenous infusion of the monoclonal antibody CIS43LS was safe and protective against *P. falciparum* malaria infection in healthy adults in Mali over an intense 6-month malaria season. This was the first time a monoclonal antibody was shown to prevent malaria infection in an endemic area.

## What’s the next step for this research?

As is often the case with new investigational drugs, we started our studies of this monoclonal antibody with healthy, non-pregnant adults, but it’s young children and pregnant women who suffer the most from malaria in Africa. So, this first proof-of-concept study in adults provided a foundation for our ongoing trials of monoclonal antibodies as we age and de-escalate to younger children. For example, we just completed a study in Mali that involved children aged 6-10 years who received a single subcutaneous dose of an anti-malarial monoclonal antibody before the 6-month malaria season. We also have an ongoing trial with colleagues in Western Kenya that involves infants and children aged 5 months to 5 years to test the safety and efficacy of subcutaneous administration of an anti-malarial monoclonal antibody in a region of intense year-round malaria transmission. If the results of these phase 2 trials look good, we’ll move on to a larger phase 3 trial to assess the safety and efficacy of an anti-malarial monoclonal antibody in infants and children, to see if and how it could add to the current arsenal of malaria prevention tools.

## What is the current standard of care?

The World Health Organization (WHO) recommends the newly approved RTS,S malaria vaccine for children 5–17 months of age living in areas of moderate to high malaria transmission. The four-shot vaccine series provides approximately 35% protective efficacy over four years. In addition, the WHO recommends malaria chemoprevention for children at high risk of malaria. For example, where malaria transmission is seasonal, the WHO recommends a 3-day course of anti-malarial drugs every month during the four-to-six-month malaria season for children under 5 years of age. These are critically important interventions to reduce the number of malaria cases and deaths, but their efficacy is not optimal, and they are labor-intensive to implement. A single dose of a monoclonal antibody before each malaria season could potentially complement or even replace monthly chemoprevention and provide more efficient protection with fewer contacts with the health system.

## That sounds incredibly motivating

I consider myself lucky to be in a position at this stage of my career working on something that has real potential to save lives. Because of our research network in Africa, we can do these studies relatively efficiently to further develop this promising new malaria prevention tool, so I wake up each day very motivated to continue this work.

## What traits are most important for someone considering a career in clinical research?

As with many careers, clinical research has its challenges, and setbacks often happen, so you need patience, perseverance, and the ability to keep the long view in mind. You need to understand that if you work through the challenges, you’ll find a way through. It’s also critical to keep an open mind and remain teachable, and to have enough humility to be able to admit when you’re wrong and change course if needed. It’s also important to listen to others and be flexible in your thinking. This is especially important if you’re interested in collaborative work across borders and cultures.

## Has the way you collaborate across borders changed over the years?

Yes and no. One of the most notable changes has been improvements in the way we communicate on a near-daily basis. Much of our day-to-day work – reviewing data, going over new protocols, etc. – is done via email and videoconferencing. Even so, I would say there is no substitute for spending time with colleagues in Mali at our field sites. It’s face-to-face time in Mali discussing ongoing and future projects that have made our collaborative work so successful. More and more, my time in Mali is also spent discussing how we can accelerate the expansion of research capacity and training in Africa, including improving laboratory and clinical infrastructure and enhancing information technology capabilities. Our collaboration has expanded over the years, and I feel very grateful to be a part of this team effort.

